# Effect of indobufen vs. aspirin on platelet accumulation in patients with stable coronary heart disease after percutaneous coronary intervention: An open-label crossover study

**DOI:** 10.3389/fphar.2022.950719

**Published:** 2022-08-16

**Authors:** Qiu-Ping Shi, Xing-Yu Luo, Bin Zhang, Xin-Gang Wang, Jing Zhao, Qiu-Fen Xie, Jia-Hui Liu, Yao-Kun Liu, Jie Jiang, Bo Zheng

**Affiliations:** ^1^ Peking University First Hospital, Beijing, China; ^2^ Institute of Cardiovascular Disease, Peking University First Hospital, Beijing, China

**Keywords:** indobufen, platelet aggregation rate, antiplatelet therapy, coronary heart disease, percutaneous coronary intervention

## Abstract

**Purpose:** This study compared the effect of indobufen with that of aspirin on platelet function in patients with stable coronary heart disease after percutaneous coronary intervention (PCI).

**Methods:** Patients with stable coronary heart disease who had undergone PCI and received dual antiplatelet therapy (aspirin 100 mg + clopidogrel 75 mg once daily) for at least 12 months were allocated to receive indobufen 100 mg twice daily + clopidogrel 75 mg once daily, clopidogrel 75 mg once daily alone, indobufen 100 mg twice daily alone, and aspirin 100 mg once daily alone for 1 month each in an open-label crossover manner. Platelet function was assessed by using the rates of arachidonic acid (AA)-induced platelet aggregation (AA-PAR) and adenosine diphosphate (ADP)-induced platelet aggregation (ADP-PAR) measured by light transmission aggregometry, the platelet reactivity index measured by vasodilator-stimulated phosphoprotein (PRI-VASP), and the plasma and urinary thromboxane B_2_ (TXB_2_) concentrations recorded at baseline and during each treatment phase.

**Results:** Of 56 patients enrolled, 52 completed the study. The AA-PAR was lower in the indobufen alone group than in the aspirin alone group [5.21% (3.39, 7.98) vs. 5.27% (4.06, 6.60), *p* = 0.038], while biologically, a difference of 0.06% may represent no significant difference; there was no significant between-group difference in the plasma [531.16 pg/ml (203.89, 1035.06) vs. 373.93 pg/ml (194.04, 681.71), *p* = 0.251] or urinary [3951.97 pg/ml (2006.95, 6077.01) vs. 3610.48 pg/ml (1664.60, 6247.61), *p* = 0.717] TXB_2_ concentration. When the aspirin + clopidogrel group and indobufen + clopidogrel group were compared, similar results were found for AA-PAR [3.97% (3.05, 5.12) vs. 3.83% (3.10, 5.59), *p* = 0.947] and both plasma [849.47 pg/ml (335.96, 1634.54) vs. 455.41 pg/ml (212.47, 1489.60), *p* = 0.629], and urinary [4122.97 pg/ml (2044.96, 7459.86) vs. 3812.81 pg/ml (1358.95, 6021.07), *p* = 0.165] TXB_2_ concentrations. ADP-PAR was lower in the clopidogrel alone group than in the indobufen alone group (47.04% ± 16.89 vs. 61.7% ± 10.50, *p* < 0.001), as was PRI-VASP (66.53% ± 18.06 vs. 77.72% ± 19.87, *p* = 0.002).

**Conclusion:** These findings suggest that indobufen has antiplatelet effects similar to those of aspirin in patients with stable coronary heart disease after PCI, and may be an alternative for patients with aspirin intolerance after coronary stenting.

## Introduction

Platelet activation and aggregation play a key role in the occurrence and development of atherosclerotic thrombosis (Davì and Patrono, 2007). Current guidelines recommend dual antiplatelet therapy combined with aspirin (acetylsalicylic acid) and an adenosine diphosphate (ADP) receptor antagonist for at least 6–12 months after percutaneous coronary intervention (PCI) followed by long-term maintenance on aspirin as antiplatelet monotherapy (Collet et al., 2021). This strategy is recommended for secondary prevention of thrombotic events, which can effectively reduce the risk of cardiovascular death, recurrent myocardial infarction and stroke and may improve the prognosis (Degrauwe et al., 2017).

Aspirin inhibits arachidonic acid (AA)-mediated platelet aggregation by irreversibly inhibiting the cyclo-oxygenase (COX) enzyme and is the cornerstone of primary and secondary prevention therapy for coronary artery disease (Antithrombotic Trialists’ Collaboration, 2002). However, aspirin is a major cause of iatrogenic gastrointestinal injury, including ulceration, erosion of the stomach, and upper gastrointestinal bleeding (Levy, 1974; Ibáñez et al., 2006), due in part to inhibition of synthesis of cytoprotective prostaglandins in the gastric mucosa (Miller and Jacobson, 1979). Furthermore, a proportion of patients are intolerant of aspirin, which can manifest as bronchospasm, urticaria/angioedema, or anaphylaxis (Stevenson, 2004), and are at high risk of discontinuation. Aspirin desensitization therapy has been found to be safe and may be effective (Bianco et al., 2016) but further confirmatory randomized controlled trials are needed. Therefore, alternative antiplatelet agents are required for this population.

Indobufen, a phenylbutyric acid derivative (Müller, 1991; Morocutti et al., 1997), is an antiplatelet agent that inhibits production of thromboxane and COX-dependent aggregation of platelets by reversible inhibition of COX-1 with less affecting production of prostacyclins. It can also reduce platelet adhesion and inhibit platelet aggregation induced by ADP (Bhana and McClellan, 2001). Previous studies have shown that indobufen is well tolerated, associated with a low incidence of adverse effects (Wiseman et al., 1992; Marzo et al., 2004), and has biochemical, functional, and clinical effects that are comparable with those of a standard dose of aspirin (Müller, 1991). In a group of patients with stable ischemia and aspirin intolerance undergoing coronary stent implantation, combined treatment with indobufen and thienopyridine resulted in a low rate of ischemic events (Latib et al., 2013). Another study suggested that combined antiplatelet treatment with clopidogrel + indobufen could be a good option in patients who are undergoing coronary stenting for acute coronary syndrome (ACS) and have aspirin hypersensitivity (Barillà et al., 2013). However, the evidence as to whether indobufen and aspirin have the same inhibitory effect on platelet aggregation has been inconsistent (De Caterina et al., 1996; Cipollone et al., 1997; Barillà et al., 2013; Lee et al., 2016; Yang et al., 2021). Recently, Yang et al. (2021) found significantly less suppression of AA-induced platelet aggregation in patients with coronary atherosclerosis who received indobufen 100 mg twice daily than in those who received aspirin 100 mg once daily but reported that both agents inhibited urinary 11-dehydrothromboxane B_2_ to a similar degree. Therefore, it remains uncertain whether the antiplatelet effect of indobufen is comparable with that of aspirin.

Few studies have used platelet function tests to compare the antiplatelet effect of indobufen with that of aspirin in patients with stable coronary heart disease after PCI. The aim of this study was to compare the effects of indobufen on platelet function with those of aspirin when used alone and in combination with clopidogrel to provide more evidence regarding whether indobufen can be used as an alternative to aspirin.

## Methods

The study protocol was approved by the Ethics Committee of Peking University First Hospital (approval number 2018-12) and performed in accordance with the tenets of the Declaration of Helsinki. Informed consent was obtained from each study participant.

### Subjects

Patients with stable coronary heart disease who had undergone PCI at Peking University First Hospital and received aspirin 100 mg + clopidogrel 75 mg once daily for at least 12 months were recruited. All patients received standard medication for secondary prevention of coronary heart disease.

Patients were considered eligible for enrolment if they met the following criteria: able to sign an informed consent form; age 18–85 years; confirmed stable coronary heart disease after PCI; and currently receiving antiplatelet therapy in combination with aspirin 100 mg + clopidogrel 75 mg once daily. The following exclusion criteria were applied: ACS within the 12 months before screening; PCI within the 12 months before screening; receiving oral or intravenous anticoagulant therapy for another condition, such as atrial fibrillation, pulmonary embolism, lower limb venous thrombosis, or an artificial heart valve; an AA-induced platelet aggregation rate (AA-PAR) measured by light transmission aggregometry (LTA) to be more than 20% when treated with aspirin + clopidogrel in the previous 3 months; congestive heart failure or left ventricular ejection fraction <35%; forced expiratory volume in 1 s or forced vital capacity below the lower reference limit; bleeding tendency or severe lung disease; active pathological bleeding; history of intracranial hemorrhage; allergy to an indobufen formulation or any of its excipients; severe liver injury (elevation of transaminases up to 3-fold the upper reference limit); pregnancy, lactation, or planning pregnancy; hematological disease, a platelet count <100,000/mm^3^, or hemoglobin <10 g/dl; glycosylated hemoglobin >10%; history of drug abuse or alcoholism in the previous 2 years; use of non-steroidal anti-inflammatory drugs; and a creatinine clearance rate <30 ml/min.

### Study interventions

**FIGURE 1:**
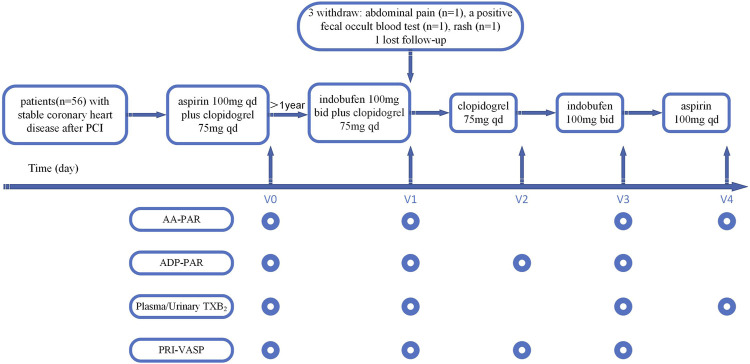
Study flow chart. AA-PAR = rate of arachidonic acid-induced platelet aggregation measured by light transmission aggregometry; ADP-PAR = rate of adenosine diphosphate-induced platelet aggregation measured by light transmission aggregometry; bid = twice daily; PCI = percutaneous coronary intervention; PRI-VASP = platelet reactivity index measured by vasodilator-stimulated phosphoprotein; qd = once daily; TXB_2_ = thromboxane B_2_; V0 = visit at baseline; V1 = visit at the first month; V2 = visit at the second month; V3 = visit at the third month; V4 = visit at the fourth month.

Patients received sequential antiplatelet therapy consisting of indobufen 100 mg twice daily + clopidogrel 75 mg once daily for 1 month (V1), clopidogrel 75 mg once daily alone for 1 month (V2), indobufen 100 mg twice daily alone for 1 month (V3), and aspirin 100 mg once daily alone for 1 month (V4) in an open-label crossover manner (Figure 1). Platelet activity and adverse events were assessed at baseline (V0) and at the end of each treatment phase (V1, V2, V3, and V4) by laboratory investigations and clinical evaluation.

We measured platelet aggregation in response to AA and ADP using LTA. Vasodilator-stimulated phosphoprotein (VASP) levels were used to measure the platelet reactivity index (PRI-VASP, %) in response to ADP. Plasma and urinary TXB_2_ concentrations were used to reflect the plasma TXA_2_ level produced by platelets after being induced by AA. Platelet function tests were performed according to the antiplatelet agent(s) used in each phase (Figure 1).

Laboratory investigations, including a whole blood count and classification, routine urine and stool tests, a fecal occult blood test, coagulation function, and serum creatinine, alanine aminotransferase, and aspartate aminotransferase levels, were performed at the start and end of the study to evaluate the safety of the trial medications. Compliance and adverse events were assessed at each follow-up visit. Other non-steroidal anti-inflammatory drugs and non-investigational antiplatelet agents, such as glycoprotein IIb/IIIa receptor antagonists, were prohibited during the study period. The indobufen used in the study was provided as 200 mg tablets free of charge by Hangzhou Zhongmei Huadong Pharmaceutical Co., Ltd. (Hangzhou, China). Aspirin was purchased as 100 mg tablets from Bayer AG (Leverkusen, Germany), and clopidogrel as 75 mg tablets from Sanofi Pharmaceuticals (Paris, France). All the trial medications were commercially available with approved labelling.

### Measurement of platelet function

The platelet aggregation rate (PAR, %) in response to AA and ADP was measured by an LTA device (Model 700, Chrono-Log Corporation, Havertown, PA, United States), and the results are reported as AA-PAR and ADP-PAR. All tests were performed by technicians in the Laboratory Department of Peking University First Hospital using standard operating procedures (Tang et al., 2015).

VASP was assessed in accordance with the standard protocol with labeled monoclonal antibodies by flow cytometry using the Platelet VASP-FCM kit (BioCytex Inc., Marseille, France) as previously described (Rollini et al., 2016). The results are reported as the platelet reactivity index (PRI-VASP, %), which was calculated after measuring VASP-P levels following stimulation with prostaglandin (PG) E1 (MFIc_PGE1_) and PGE1 + ADP (MFIc_PGE1 + ADP_). PRI was calculated as [(MFIc_PGE1_) − (MFIc_PGE1 + ADP_)/(MFIc_PGE1_)] × 100%.

Plasma and urinary TXB_2_ concentrations were measured using the Thromboxane B_2_ ELISA Kit-Monoclonal (Item No. 501020, Cayman Chemical, Ann Arbor, MI, United States) in accordance with the manufacturer’s instructions and confirmed using standard curves (R2 > 99%).

### Study endpoints

The primary study endpoint was the difference in AA-PAR between the indobufen alone group and the aspirin alone group. The secondary endpoints were as follows: 1) differences in plasma and urinary TXB_2_ concentrations between the indobufen alone group and the aspirin alone group; 2) differences in AA-PAR and in plasma and urinary TXB_2_ concentrations between the aspirin + clopidogrel group and the indobufen + clopidogrel group; and 3) differences in ADP-PAR and PRI-VASP between the clopidogrel alone group and the indobufen alone group. Patient adherence to the antiplatelet regimen was monitored during follow-up, and all adverse events were recorded.

### Acquisition of data and statistical analysis

A case report form was used for data collection and questioning purposes. Data were entered in duplicate into an Epidata database by two administrators working independently. Continuous data were summarized as the mean ± standard deviation or the median (interquartile range) and compared using the t-test and paired t-test as appropriate. Categorical variables are presented as the count and proportion (percent), and they were compared using the chi-squared and McNemar’s chi-squared test as appropriate. The statistical analysis was performed using EmpowerStats software (www.empowerstats.com) and SPSS 24.0 software. A two-sided *p*-value < 0.05 was considered statistically significant.

## Results

Fifty-six patients were enrolled in the study. All 52 patients who completed the study had undergone PCI for stable coronary heart disease and had received aspirin 100 mg + clopidogrel 75 mg once daily for at least 12 months. Table 1 shows the patient demographics, clinical characteristics, and medications used at baseline. Four patients dropped out of the study before visit 1 because of abdominal pain (*n* = 1), a positive fecal occult blood test (*n* = 1), rash (*n* = 1), or an unknown reason (*n* = 1).

**TABLE 1:** Patient demographics and clinical characteristics.

Characteristics	Values (*n* = 56)
Age (years)	63.07 ± 6.46
BMI (kg/m^2^)	25.31 ± 2.62
Male (n, %)	54 (96.43)
Hypertension (n, %)	33 (58.93)
Diabetes (n, %)	26 (46.43)
Hyperlipidemia (n, %)	52 (92.86)
Prior ACS (n, %)	52 (92.86)
Prior PCI (n, %)	56 (100)
Concomitant medication (n, %)	
Aspirin	56 (100)
Clopidogrel	56 (100)
Statin	54 (96.4)
β-blocker	38 (67.9)
ACEI/ARB	26 (46.4)
CCB	15 (26.8)
Nitrate	14 (25.0)
Ezetimibe	15 (26.8)
Trimetazidine	9 (16.1)
Nicorandil	4 (7.1)
Oral hypoglycemic drugs	20 (35.7)
Insulin	5 (8.9)
PPI	10 (17.9)

Data are shown as the mean ± standard deviation or the number (percentage). ACEI, angiotensin-converting enzyme inhibitor; ACS, acute coronary syndrome; ARB, angiotensin receptor blocker; BMI, body mass index; CCB, calcium channel blocker; PCI, percutaneous coronary intervention; PPI, proton pump inhibitor.

### Platelet aggregation

**FIGURE 2:**
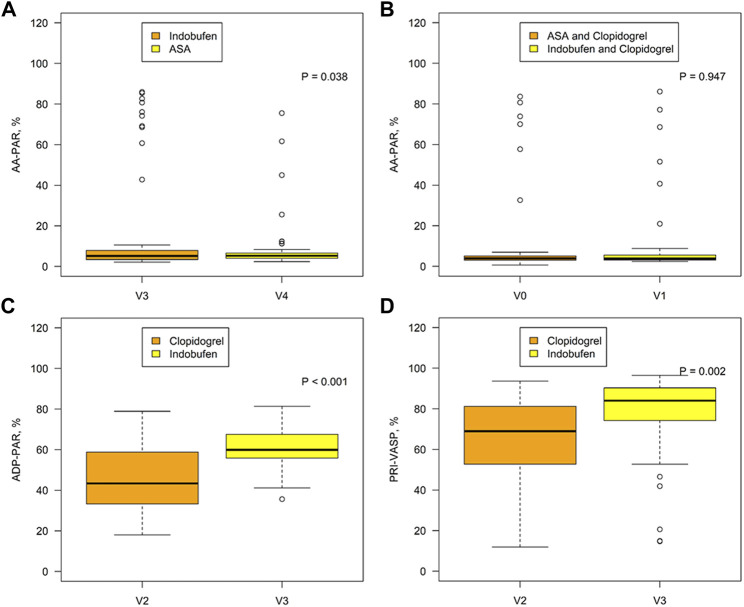
Comparison of the AA-PAR between the indobufen alone group (V3) and the aspirin alone group (V4) **(A)** and between the aspirin + clopidogrel group (V0) and the indobufen + clopidogrel group (V1) **(B)**. Comparison of the ADP-PAR **(C)** and PRI-VASP **(D)** between the clopidogrel alone group (V2) and the indobufen alone group (V3). AA-PAR = rate of arachidonic acid-induced platelet aggregation; ADP-PAR = rate of adenosine diphosphate-induced platelet aggregation; PRI-VASP = platelet reactivity index measured by vasodilator-stimulated phosphoprotein.

AA-PAR was found to be lower in the indobufen alone group than in the aspirin alone group [5.21% (3.39, 7.98) vs. 5.27% (4.06, 6.60), *p* = 0.038; Figure 2A] but was similar between the aspirin + clopidogrel group and indobufen + clopidogrel group [3.97% (3.05, 5.12) vs. 3.83% (3.10, 5.59), *p* = 0.947; Figure 2B]. ADP-PAR was lower in the clopidogrel alone group than in the indobufen alone group (47.04% ± 16.89 vs. 61.7% ± 10.50, *p* < 0*.*001), as was the PRI-VASP value (66.53 ± 18.06 vs. 77.72 ± 19.87, *p* = 0.002, Figure 2C).

### Plasma and urinary TXB_2_ concentrations

**FIGURE 3:**
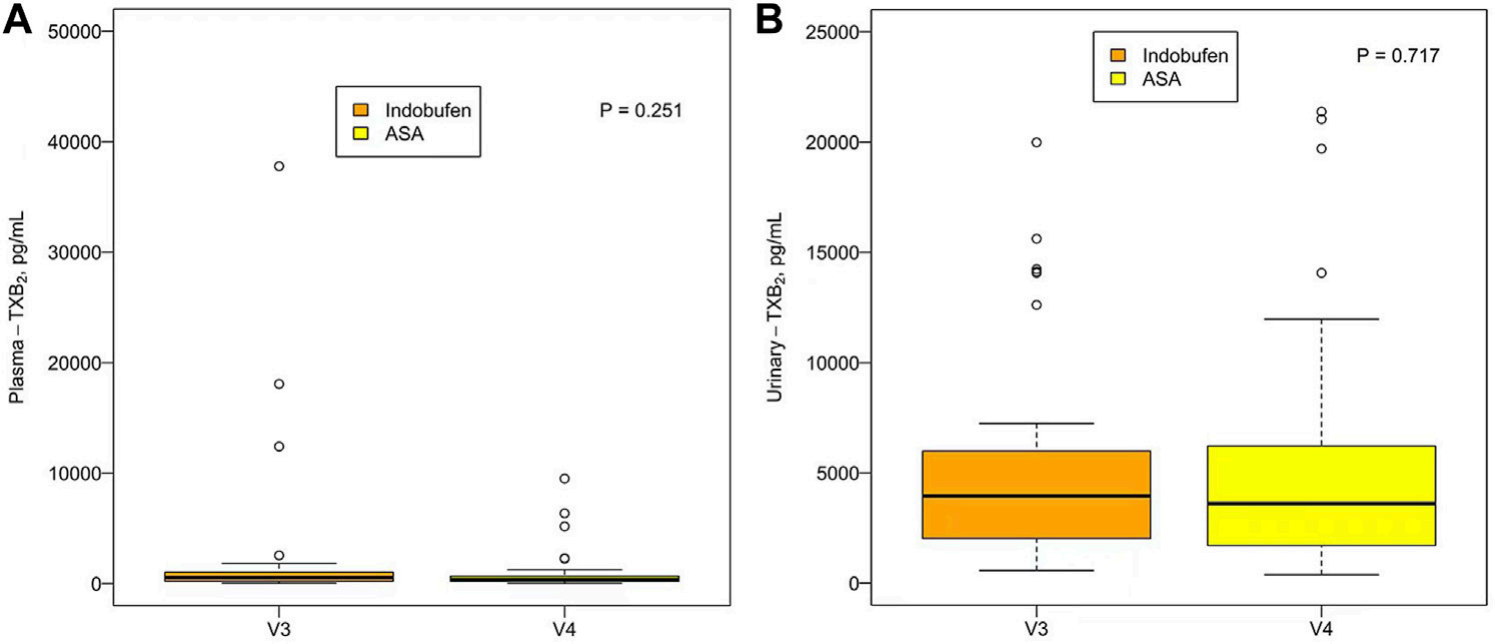
**(A)** Comparison of the **(A)** plasma and **(B)** urinary TXB_2_ concentration between the indobufen alone group (V3) and the aspirin alone group (V4). ASA = aspirin; TXB_2_ = thromboxane B_2_.

**FIGURE 4:**
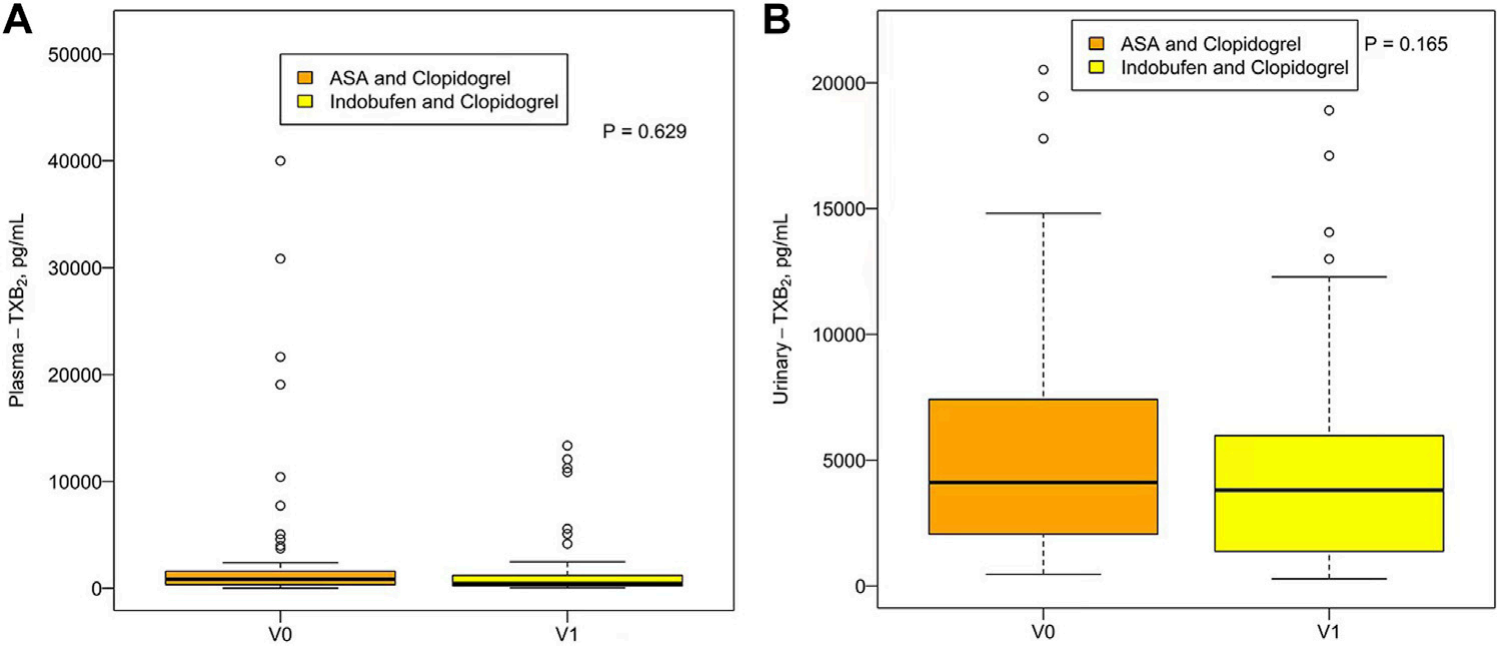
Plasma **(A)** and urinary **(B)** TXB_2_ concentrations in the aspirin + clopidogrel group (V0) and the indobufen + clopidogrel group (V1). ASA = aspirin; TXB_2_ = thromboxane B_2_.

There was no significant difference in plasma TXB_2_ concentration between indobufen and aspirin [531.16 pg/ml (203.89, 1035.06) vs. 373.93 pg/ml (194.04, 681.71), *p* = 0.251] or urinary TXB_2_ concentration [3951.97 pg/ml (2006.95, 6077.01) vs. 3610.48 pg/ml (1664.60, 6247.61), *p* = 0.717; Figures 3A,B]. The plasma and urinary TXB_2_ concentrations were similar in the aspirin + clopidogrel group and the indobufen + clopidogrel group [849.47 pg/ml (335.96, 1634.54) vs. 455.41 pg/ml (212.47, 1489.60), *p* = 0.629 and 4122.97 pg/ml (2044.96, 7459.86) vs. 3812.81 pg/ml (1358.95, 6021.07), *p* = 0.165; Figures 4A,B].

### Clinical outcomes

There was no significant difference in blood pressure, pulse rate, or laboratory test results between baseline and the end of the study (Supplementary Tables S1, S2). According to the Bleeding Academic Research Consortium consensus document (Mehran et al., 2011), there were no severe bleeding events during the study that met the criteria for type 2, 3, 4, or 5, including fatal or life-threatening bleeding, a clinically significant or obvious bleeding-related decrease in hemoglobin, or need for blood transfusion. Gastrointestinal discomfort and bleeding events that occurred during the study period are shown in Table 2. Two minor type 1 bleeding episodes were noted at visit 1, one in a patient who had a positive fecal occult blood test that became negative on re-examination and the other in a patient who developed mild epistaxis that recovered spontaneously. There were no serious or life-threatening adverse events that resulted in hospitalization, disability, dysfunction, deformity, or death.

**TABLE 2:** Bleeding events and gastrointestinal discomfort during the study period.

Events	Visit 1	Visit 2	Visit 3	Visit 4
	Indobufen + clopidogrel	Clopidogrel	Indobufen	Aspirin
Type 1 bleeding	2^a^	0	0	0
Type 2 bleeding	0	0	0	0
Type 3 bleeding	0	0	0	0
Type 4 bleeding	0	0	0	0
Type 5 bleeding	0	0	0	0
Gastrointestinal discomfort	4	0	2	1

a Epistaxis and positive occult fecal blood, respectively.

Bleeding classification according to the Bleeding Academic Research Consortium criteria.

## Discussion

To our knowledge, this is the first study to compare the antiplatelet effect of indobufen with that of aspirin in patients with stable coronary heart disease after PCI using platelet function testing in both monotherapy and dual antiplatelet therapy scenarios. The major findings of this study were as follows: 1) AA-PAR was similar in the indobufen alone group with that in the aspirin alone group, though the *p* value was less than 0.05, a difference of 0.06% may represent no significant difference biologically; 2) there was no significant difference in the plasma or urinary TXB_2_ concentration between the indobufen alone group and the aspirin alone group; 3) there were no significant differences in AA-PAR or plasma or urinary TXB_2_ concentrations between the indobufen + clopidogrel group and the aspirin + clopidogrel group; 4) ADP-PAR and PRI-VASP values were higher in the indobufen alone group than in the clopidogrel alone group.

A platelet function test can reflect individual responsiveness to antiplatelet agents and help with formulation of antiplatelet treatment strategies. The methods commonly used to detect platelet function include LTA, the VerifyNow system, thromboelastography, flow cytometry to detect platelet VASP phosphorylation levels, and the plasma TXB_2_ level (Paniccia et al., 2015).

Indobufen and aspirin exert their antiplatelet effects mainly by inhibiting COX-1, thereby inhibiting synthesis of TXA_2_ and aggregation of platelets (Eikelboom et al., 2002). Therefore, we can assess their antiplatelet efficacy *via* the indirect effect of TXA_2_-induced platelet aggregation by adding AA to blood samples (Morocutti et al., 1997). LTA, developed by Born in the 1960s, is performed using platelet-rich plasma as the milieu. By adding a variety of agonists such as AA or ADP to platelet-rich plasma, the corresponding residual platelet response rate can be obtained and is usually expressed as the PAR. It is the most widely employed methodology for detecting disorders of platelet function and monitoring the effects of antiplatelet therapy. The residual platelet reactivity rate defined by ADP-LTA, AA-LTA, or both, has been associated with ischemic events in patients with ACS and in those with stable coronary artery disease (Breet et al., 2010). Furthermore, TXA_2_ is rapidly transformed by hydrolysis into TXB_2_, which is a biologically inactive and stable product (Wiseman et al., 1992; Latib et al., 2013). Serum and urinary TXB_2_ metabolites reflect biosynthesis of TXA_2_ and are useful for assessment of platelet function in various disease states, detection of defects in production of thromboxane, and monitoring the effects of antiplatelet therapy.

Previous studies have used platelet function to compare the antiplatelet effect of indobufen with that of aspirin. In 1996, De Caterina et al. (1996) showed that aspirin (300 mg/day for 1 week) and indobufen (200 mg twice a day for 1 week) reduced the plasma TXB_2_ level and inhibited the maximum extent of whole blood platelet aggregation to similar extents in patients with ischemic heart disease and in healthy volunteers. Cipollone et al. (1997) reported that urinary excretion of 11-dehydrothromboxane B_2_ was significantly lower in patients with unstable angina who received indobufen (200 mg twice a day) than in their counterparts who received aspirin (320 mg daily). Moreover, a study by Barillà et al. (2013) that included 42 consecutive patients with ACS and hypersensitivity to aspirin undergoing coronary stenting found that the maximum percent platelet aggregation in response to AA was lower in those who received clopidogrel 75 mg daily + indobufen 100 mg twice a day than in those who received clopidogrel 75 mg daily alone. In the study by Barillà et al., the plasma TXB_2_ level at 1 week and 1 month was also very low in the patients whose treatment included indobufen.

In a study by Lee et al. (2016) in which 20 healthy volunteers received aspirin (200 mg/day for 2 weeks) followed by a 4-week washout period and then indobufen (200 mg twice a day for 2 weeks), the percent inhibition of platelet aggregation assessed using AA as the agonist was similar at 4 h after the last dose of indobufen and aspirin but was significantly lower after the last dose of indobufen than after the last dose of aspirin at 12, 24, and 48 h.

All the above-mentioned studies showed that the inhibitory effect of indobufen on platelet aggregation was at least equivalent to that of aspirin and that the anti-aggregation effect diminished more rapidly after indobufen than after aspirin. However, as mentioned earlier, the results of the study by Yang et al. (2021) were different in that AA-induced platelet aggregation was significantly less suppressed in patients with coronary atherosclerosis who received indobufen 100 mg twice daily than in those who received aspirin 100 mg once daily. Furthermore, Yang et al. reported that the inhibitory effect on the plasma TXB_2_ level in healthy volunteers at 8 and 12 h after the final dose of indobufen was weaker than that after the final dose of aspirin. Therefore, more studies are needed to confirm whether the antiplatelet effect of indobufen is comparable with that of aspirin.

The present study had an open-label crossover design whereby each patient served as their own matched control to minimize the influence of interindividual differences in drug metabolism. Furthermore, the study population comprised patients with stable coronary heart disease after PCI; thus, our results could be used to guide antiplatelet therapy in patients with coronary heart disease. In terms of dose selection, in the Chinese population, the more likely dosage of indobufen would be 100 mg twice a day (Author Anonymous, 2019). Therefore, in our study, we chose this dosage for evaluation.

Our main finding in this study was that indobufen 100 mg twice daily inhibited AA-induced platelet aggregation detected by the LTA method to a significantly greater extent than aspirin 100 mg once daily, though a difference of 0.06% may represent no significant difference biologically, with no significant between-group difference in the plasma or urinary TXB_2_ concentration. Moreover, there was no difference in the platelet aggregation rate or thromboxane concentration between the indobufen and aspirin groups when clopidogrel was added. Overall, we found that the antiplatelet effects *via* the AA pathway were similar between indobufen and aspirin.

Like clopidogrel, indobufen can inhibit platelet aggregation induced by ADP Bianco et al. (2016). In an *in vitro* and *in vivo* study, Li et al. (2021) showed that indobufen + clopidogrel had a higher inhibitory effect on ADP induced platelet aggregation than aspirin + clopidogrel. However, in the study by Barillà et al. (2013), there was no difference in the maximum inhibition rate of ADP-induced aggregation of platelets between the group that received clopidogrel 75 mg daily + indobufen 100 mg twice a day and the group that received clopidogrel 75 mg daily alone, suggesting that indobufen does not increase the inhibition of ADP-induced platelet aggregation further.

To date, few studies have compared the antiplatelet effects of indobufen alone with those of clopidogrel alone. Among the currently available methods used to assess platelet function, the most established and clinically validated ones used to explore ADP-induced platelet aggregation are VerifyNow P2Y12, LTA, and VASP (Rollini et al., 2016). In our study, we performed LTA and VASP assays to compare the ability of indobufen to inhibit platelet aggregation with that of clopidogrel. The PRI-VASP is used to assess P2Y12 receptor blockade and is specific and reproducible. This test is highly specific for the P2Y12 receptor pathway and correlates with the concentration of active metabolites. According to the PRI, platelet reactivity is divided into low (LPR), optimal (OPR), or high (HPR), and the respective cut-off values for the LPR, OPR, and HPR categories are <16%, 16%–50%, and >50%. Several studies have found that HPR is associated with a greater risk of ischemic complications while LPR has been associated with a greater likelihood of bleeding events (Bonello et al., 2012; Tantry et al., 2013; Aradi et al., 2014; Aradi et al., 2015). In our study, both ADP-PAR and PRI-VASP were higher in the indobufen alone group than in the clopidogrel alone group. PRI-VASP was 77.72% ± 19.87% in the indobufen group and 66.53% ± 18.06% in the clopidogrel group. Therefore, our study also suggests that indobufen inhibits platelet aggregation by inhibiting the ADP pathway but not as well as clopidogrel.

A more relevant issue was to look at non-response to the anti-platelet agents. In LTA non-response to AA in aspirin-treated patients is usually set at 20% (Hankey and Eikelboom, 2006). There were five non-responders in aspirin plus clopidogrel group and six non-responders in indobufen plus clopidogrel group (Supplementary Table S3), and the chi-square test showed that there was no significant difference between the two groups (*p* = 1.000). There were 10 non-responders in indobufen alone group and four non-responders in aspirin alone group (Supplementary Table S3), and the chi-square test showed no significant difference between the two groups as well (*p* = 0.114). Non-adherence to the treatment has been previously shown to be the major cause of aspirin non-response in patients (Peace et al., 2010). Similarly, our statistical results showed that in the indobufen plus clopidogrel group and indobufen alone groups, the responders were more compliant and showed lower plasma and urinary TXB_2_ concentrations (Supplementary Table S4). It looks like that non-adherence to the indobufen may be the major cause of indobufen non-response in patients. Furthermore, there were 2, 1, 4, and 0 non-inhibiters (>80% LTA) in aspirin plus clopidogrel group, indobufen plus clopidogrel group, indobufen alone group and aspirin alone group respectively. It looks like that four of the non-responders to indobufen show no inhibition at all while none on aspirin show such high levels. Once clopidogrel is added in this removes all differences. This suggests that even with AA there is a small component of aggregation that is ADP-dependent. This is a similar finding to what was previously reported by Cox et al. (2006).

There were no serious complications requiring hospitalization or medical intervention or any life-threatening complications, including serious bleeding, throughout the study. Bleeding events and gastrointestinal discomfort occurred infrequently, which suggests that indobufen would be a safe antiplatelet therapy in patients with coronary heart disease after PCI.

Our study has several potential limitations. First, the small sample size reduced the power of the study to detect significant differences between groups. More prospective multicenter studies are needed to evaluate the efficacy and safety of indobufen in patients with stable coronary heart disease after PCI. Second, we assessed platelet function by testing the urinary TXB_2_ concentration rather than urinary 11-dehydrothromboxane B_2_, which is the major product of TXB_2_ found in urine and provides a better indirect assessment of the ability of platelets to form TXA_2_. Third, the open-label design of this study introduces the possibility of bias, even though the adjudication of endpoints was performed in a blinded manner by the study evaluators. Fourth, the study was not powered for clinical outcomes. Therefore, we cannot draw any conclusions regarding the efficacy and safety of the treatment regimens used. Fifth, the most of the patients were male and it may bias the experimental results, and given that we only included Chinese patients, our results may not be generalizable to all populations. Last but not least, during the study, aspirin and clopidogrel were purchased and taken by patients themselves, so we did not recycle aspirin and clopidogrel, and lacked relevant compliance evaluation. However, we recovered the drugs after indobufen combined with clopidogrel and indobufen alone treatment, and evaluated the drug use compliance, which reached 97.69% ± 9.27% and 96.49% ± 17.48% respectively. Therefore, the overall compliance of indobufen treatment is relatively ideal. We cannot compare the compliance between indobufen treatment and non indobufen treatment, which is the limitation of this study, and we will correct it in the future study.

The findings of this study suggest that the antiplatelet effect of indobufen is equivalent to that of aspirin in patients with stable coronary heart disease after PCI. Indobufen may be an option for patients with aspirin severe hypersensitivity, resistance or intolerance after coronary stent implantation. More multicenter studies are needed to evaluate the efficacy and safety of this new antiplatelet agent in patients with coronary disease.

## Data Availability

The raw data supporting the conclusions of this article will be made available by the authors, without undue reservation.
