# Supplementary material for: Effect of indobufen vs. aspirin on platelet accumulation in patients with stable coronary heart disease after percutaneous coronary intervention: An open-label crossover study
Source: Front Pharmacol. 2022 Aug 16;13:950719. doi: 10.3389/fphar.2022.950719 (PMC9424757; doi:10.3389/fphar.2022.950719)
Supplement: Supplementary file 1 [file Table1.DOCX]

| Supplemental Table 1. Vital signs during follow-up | | | | |  |
| --- | --- | --- | --- | --- | --- |
| Vital signs | **V0** | **V1** | **V2** | **V3** | **V4** |
| SBP (mmHg) | 131.02 ± 15.24 | 131.73 ± 16.40 | 130.67 ± 15.68 | 131.52 ± 15.30 | 134.15 ± 15.21 |
| DBP (mmHg) | 77.59 ± 9.09 | 77.22 ± 9.68 | 77.84 ± 9.28 | 78.87 ± 10.05 | 77.92 ± 9.37 |
| Pulse (bpm) | 69.91 ± 11.36 | 70.93 ± 11.92 | 70.24 ± 11.38 | 71.12 ± 10.73 | 69.65 ± 12.00 |

V0, V2, V3, and V4 represent the baseline and first, second, third and fourth monthly follow-up visits, respectively. DBP = diastolic blood pressure; SBP = systolic blood pressure
