# Supplementary material for: Effect of indobufen vs. aspirin on platelet accumulation in patients with stable coronary heart disease after percutaneous coronary intervention: An open-label crossover study
Source: Front Pharmacol. 2022 Aug 16;13:950719. doi: 10.3389/fphar.2022.950719 (PMC9424757; doi:10.3389/fphar.2022.950719)
Supplement: Supplementary file 2 [file Table2.DOCX]

| \| Supplemental Table 2. Results of laboratory investigations \| \| --- \| | | | |
| --- | --- | --- | --- | --- |
| Parameter | **V0 (n=56)** | **V4 (n=52)** | ***p-*value** |
| RBC (*10^12) | 4.73 ± 0.51 | 4.84 ± 0.50 | 0.432 |
| HGB (g/L) | 147.20 ± 14.33 | 150.10 ± 13.70 | 0.397 |
| PLT (*10^9/L) | 195.11 ± 44.50 | 196.75 ± 45.80 | 0.741 |
| Scr (µmol/L) | 84.72 ± 15.43 | 85.11 ± 16.72 | 0.956 |
| ALT (IU/L) | 22.64 ± 10.30 | 25.75 ± 12.36 | 0.203 |
| AST (IU/L) | 22.70 ± 6.81 | 24.48 ± 7.15 | 0.190 |
| PT (s) | 10.99 ± 0.55 | 10.97 ± 0.65 | 0.822 |
| APTT (s) | 30.63 ± 3.30 | 30.93 ± 3.27 | 0.590 |
| FOB | 0 (32, 100%) | 0 (33, 100%) | 0.808 |

Normally distributed data are presented as the mean ± standard deviation. Categorical data are presented as the number (percentage).

ALT = alanine aminotransferase; APTT = activated partial thromboplastin time; AST = aspartate aminotransferase; FOB = fecal occult blood; HGB = hemoglobin concentration; PLT = platelet count; PT = prothrombin time; RBC = red blood cell count; Scr = serum creatinine
