# Supplementary material for: Effect of indobufen vs. aspirin on platelet accumulation in patients with stable coronary heart disease after percutaneous coronary intervention: An open-label crossover study
Source: Front Pharmacol. 2022 Aug 16;13:950719. doi: 10.3389/fphar.2022.950719 (PMC9424757; doi:10.3389/fphar.2022.950719)
Supplement: Supplementary file 3 [file Table3.DOCX]

**Supplemental Table 3. Display of responsive and non-responsive patients**

The McNemar's chi-square test was used to statistically analyze the differences between groups.

V0 = aspirin plus clopidogrel group; V1 = indobufen plus clopidogrel group; v3 = indobufen alone group; v4 = aspirin alone group

| Group | n | Responder, (n,%) | Non-responder, (n,%) | McNemar's chi-squared | *p*-value |
| --- | --- | --- | --- | --- | --- |
| V0 | 52 | 47 (90.38%) | 5 (9.62%) | 0.000 | 1.000 |
| V1 | 52 | 46 (88.46%) | 6 (11.54%) |  |  |
| V3 | 50 | 40 (80.00%) | 10 (20.00%) | 2.500 | 0.114 |
| V4 | 50 | 46 (92.00%) | 4 (8.00%) |  |  |
