# Supplementary material for: Effect of indobufen vs. aspirin on platelet accumulation in patients with stable coronary heart disease after percutaneous coronary intervention: An open-label crossover study
Source: Front Pharmacol. 2022 Aug 16;13:950719. doi: 10.3389/fphar.2022.950719 (PMC9424757; doi:10.3389/fphar.2022.950719)
Supplement: Supplementary file 4 [file Table4.DOCX]

**Supplemental Table 4. Compliance and TXB_2_ concentrations**

Continuous data were summarized as the mean ± standard deviation or the median (interquartile range) and compared using the paired t-test as appropriate.

V1 = indobufen plus clopidogrel group; v3 = indobufen alone group; v4 = aspirin alone group

| Group | parameter | Responders | Non-responders | *p*-value |
| --- | --- | --- | --- | --- |
| V1 | Compliance (%) | 98.69 ± 7.86 | 90.02 ± 15.54 | 0.030 |
|  | Plasma TXB_2_ (pg/ml) | 418.83 (217.34,922.62) | 2345.72 (248.68,11065.20) | 0.302 |
|  | Urinary TXB_2_ (pg/ml) | 3515.42 (1258.12-5520.60) | 10969.86 (6362.27,12824.56) | 0.010 |
| V3 | Compliance (%) | 99.80 ± 9.80 | 83.54 ± 32.15 | 0.009 |
|  | Plasma TXB_2_ (pg/ml) | 313.35 (178.00,723.08) | 1383.60 (588.04,9949.21) | 0.001 |
|  | Urinary TXB_2_ (pg/ml) | 2765.07 (1970.40,5122.08) | 5932.41 (4683.51,11269.98) | 0.016 |
